# Correction to “Detection and Prediction of Neurodegenerative Disorders by Long Noncoding RNA Signature Analysis among Bangladeshi Population”

**DOI:** 10.1155/bmri/9764056

**Published:** 2026-01-31

T. Rahman, P. Rozario, M.R. Naim, F. Seraj, A.H. Chowdhury, “Detection and Prediction of Neurodegenerative Disorders by Long Noncoding RNA Signature Analysis among Bangladeshi Population,” *BioMed Research International*, 2023, https://doi.org/10.1155/2023/9416281

In the article, there are errors in the sample sizes reported in the Abstract and the Materials and Methods sections. In the abstract,

• “The purpose of this research also included the possibility of prediction of these neurodegenerative disorders by analyzing and comparing the data which was acquainted from the study where 20 PD patients and 20 healthy subjects and 25 MND patients and 25 healthy subjects of Bangladesh were examined.”

should read as follows:

• “The purpose of this research also included the possibility of prediction of these neurodegenerative disorders by analyzing and comparing the data which was acquainted from the study where 138 PD patients and 138 healthy subjects and 138 MND patients and 138 healthy subjects of Bangladesh were examined.”

In the Materials and Methods,

• “45 healthy samples were used as the control. After qRT‐PCR amplification, a melting curve analysis was performed to confirm reaction specificity, and the fold change (FC) of each lncRNA was calculated via the *Δ*Ct method.”

should read as follows:

• 276 healthy samples were used as the control. After qRT‐PCR amplification, a melting curve analysis was performed to confirm reaction specificity, and the fold change (FC) of each lncRNA was calculated via the *Δ*Ct method.

We apologize for these errors.

